# Magnetic Resonance Neurography (MRN) of the Brachial Plexus: A Case of Parsonage Turner Syndrome and a Basic Review of Imaging of the Brachial Plexus

**DOI:** 10.7759/cureus.15228

**Published:** 2021-05-25

**Authors:** Hassan Kesserwani, Adriana Faulkner

**Affiliations:** 1 Neurology, Flowers Medical Group, Dothan, USA; 2 Radiology, Radiology Associates of Dothan, Dothan, USA

**Keywords:** supraclavicular brachial plexus, mri images, brachial plexopathy, mri imaging, peripheral weakness

## Abstract

The Parsonage-Turner syndrome (PTS) or immune-mediated brachial plexopathy is a monophasic illness with well-described semiology and reasonable insights into pathogenesis. With the advent of spectacular advancements in magnetic resonance imaging (MRI) technology directed at shortening the T2 echo times and annihilating the "magic angle" and with short tau inversion recovery (STIR) sequences, we now have a new window into the evolution of inflammatory changes involving the nerve roots, brachial plexus and the peripheral nerves in inflammatory diseases of the nerves. Not only can these imaging modalities exclude other structural pathologies but they can also localise disease of the brachial plexus and outline the extent of disease and so allow the clinician to explore the natural history of immune-mediated brachial plexopathies. Indeed, these imaging sequences can antedate electromyographic findings and they can determine the effects of chronic denervation of muscle and fatty replacement. We present one such case of the PTS in order to demonstrate the power of these imaging modalities. In so doing, we outline some of the very basic correlations between the physics of MRI and pathology of the brachial plexus. An unexpected finding in this case report is the dramatic resolution of power loss following immunotherapy in our patient who had positive image findings on T2-weighted sequences and STIR imaging and who otherwise has had a static course. The implications of these findings are explored and adumbrated on.

## Introduction

Parsonage-Turner syndrome (PTS) is known by several synonyms: immune-mediated brachial plexopathy, idiopathic brachial plexitis and neuralgic amyotrophy. The clinical profile is quite characteristic - a monophasic disease heralded by typically severe shoulder-girdle pain and sometimes upper or even forearm pain, followed by variable patterns of weakness over the course of days, a static period and then gradual recovery of power over the next 6-12 months [1]. Despite immune-mediated plexopathy being the more consistent term with respect to its pathogenesis, we will adopt the term PTS due to its widespread use.

The semiology is also variable with shoulder girdle weakness being the most common pattern involving the upper trunk and posterior cord of the brachial plexus. Next in order of frequency is involvement of the lower trunk and medial cord of the brachial plexus with loss of precision grip. The least common phenotype is the "dangles" with wrist and finger drop implicating a posterior cord plexopathy. The latter is never isolated but usually part of a mixed pattern. In a study of 199 patients with immune-mediated brachial plexopathy, involvement of the upper and middle trunk with involvement of the long thoracic and/or suprascapular nerve occurred most frequently [2]. Rarer more restricted patterns such as an isolated musculocutaneous neuropathy with elbow flexion weakness [3], a phrenic nerve paralysis with hemi-diaphragm elevation [4], long thoracic nerve palsy with scapular winging [5], or an anterior interosseus nerve palsy with loss of precision grip can also occur albeit, rarely [6]. A viral syndrome may be a harbinger of PTS and rarely a frank herpetic eruption may occur, indicating post-varicella zoster segmental paresis [7]. Pathological studies demonstrating direct inflammatory inflammation of the brachial plexus are also rare [8].

The imaging profile of PTS is evolving and parallels the increased sophistication of imaging modalities. Two specific patterns have emerged in the radiological analysis of PTS. Firstly, the imaging characteristics in the acute phase of the disease appear to be quite distinct from that in the chronic phase, with hour-glass constrictions requiring surgical release recently discovered for the latter [9]. Secondly, whereas studies have initially inferred specific nerve involvement from the pattern of muscle disease (atrophy, signal changes) [10], more recent advanced radiological imaging modalities, magnetic resonance neurography (MRN), have highlighted inflammation of the nerve roots, brachial plexus trunks, divisions, cords and peripheral nerves [11].

## Case presentation

A 58-year-old right-handed woman presents with unprovoked vague tingling of the left hand and forearm, intense acute left shoulder girdle pain with rapid weakness over a few days of the left upper arm, and difficulty picking up utensils from the kitchen cabinet but with unaffected power and precision grip of the hand. By two months her disease was static and she had noted wasting of her left shoulder girdle, specifically deltoid. There was no report of a viral prodrome or rash and she has no family history of a plexopathy. Past medical history was remarkable for hypertension and depression. Medications included venlafaxine 150 milligrams (mg) daily and lisinopril 10 mg daily.

On examination, weight is 184 pounds, height is 5 feet and 7 inches, with a body-mass-index of 28.8 $kg/m^{2}$. Gait posture, stability, cadence and tandem-walking was normal. There were no cranial nerve deficits; of note, absent Horner's pupil. Power in all the limbs was perfectly normal except for the left upper limb, where the muscle strength was graded with the Medical Research Council (MRC) scale below. Profound atrophy was noted over the left deltoid muscle (Table 1).

**Table 1:** Left upper extremity muscle power grading. MRC: Medical Research Council

MUSCLE	MRC GRADE
LEFT DELTOID	2
LEFT SUPRASPINATUS	4
LEFT BICEPS	4
LEFT TRICEPS	3
LEFT BRACHIORADIALIS	5
LEFT BRACHIALIS	5
LEFT EXTENSOR CARPI RADIALIS LONGUS	5
LEFT FLEXOR CARPI ULNARIS	5
LEFT FLEXOR POLLICIS LONGUS	5
LEFT EXTENSOR DIGITORUM COMMUNIS	5
LEFT ABDUCTOR POLLICIS BREVIS	5
LEFT INTEROSSEI	5
LEFT PRONATOR TERES	5
LEFT SUPINATOR	

Deep tendon reflexes were graded at 2/2 throughout the upper and lower extremities except for an absent left biceps reflex. Sensory examination in the fingers was normal touch-pressure and pain, with normal joint position sense. The rest of the neurological examination was unremarkable.

**Figure 1:**
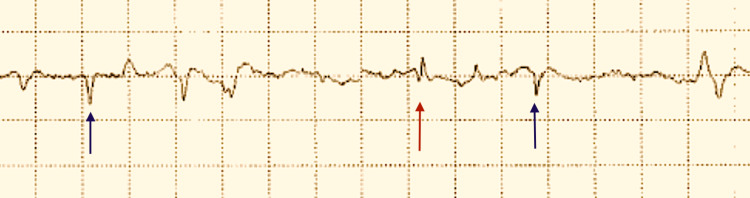
Electromyography of the left deltoid muscle demonstrating acute denervation. Fibrillation potentials (red arrow), positive waves (blue arrows).

A nerve conduction study showed preserved left median motor, ulnar motor and radial motor compound muscle action potentials and velocities. Superficial radial sensory, median sensory and ulnar sensory amplitudes and velocities were normal. An electromyogram (EMG) revealed acute denervation of the left deltoids, biceps and triceps with large long-duration polyphasic motor units with reduced recruitment and interference pattern. Acute denervation of the left deltoid is shown below (Figure 1).

**Figure 2:**
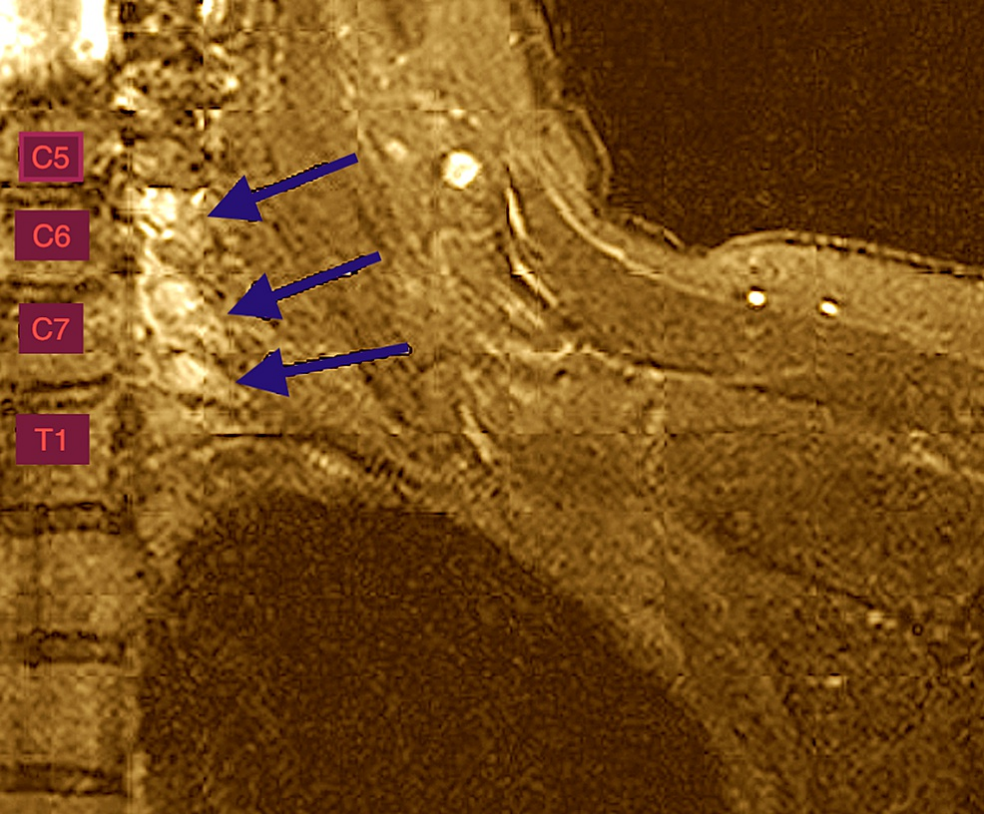
STIR-weighted coronal MRI of the brachial plexus demonstrating abnormal hyperintense signal and hypertrophy of the C6, C7 and C8 nerve roots (blue arrows). Cervical (C), Thoracic (T). STIR MRI: Short-T1 Inversion Recovery Magnetic Resonance Imaging

Acute denervation of the left deltoids and biceps is consistent with a left upper trunk plexopathy. The deltoids involvement may implicate the posterior cord of the brachial plexus and the biceps involvement may implicate the lateral cord of the brachial plexus or the musculo-cutaneous nerve, the cervical C5, C6 and C7 myotomes. A magnetic resonance imaging (MRI) study of the cervical spine reveals mild spondylosis at multiple levels. However, a short tau inversion recovery (STIR) MRI coronal image revealed hypertrophied and hyperintense cervical C6, C7 and C8 nerve roots (Figure 2).

**Figure 3:**
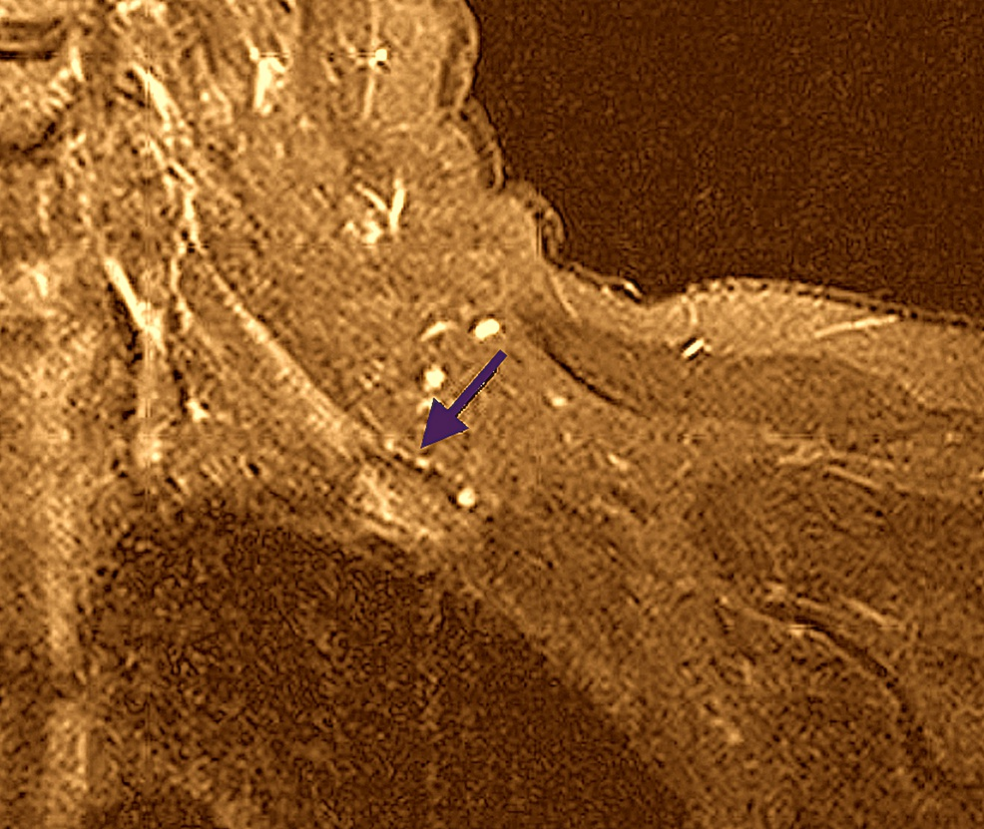
STIR-weighted coronal MRI of the brachial plexus demonstrating mildly increased signal at the level of the trunks, involving the middle trunk and portions of the upper and lower trunks (blue arrow). STIR MRI: Short-T1 Inversion Recovery Magnetic Resonance Imaging

STIR MRI images also showed hyperintensities of all the trunks of the brachial plexus (Figure 3).

Our patient was treated empirically with a three-day course of daily one-gram intravenous methylprednisolone followed by a tapering dose of prednisone over the course of two months starting at 40 mg a day. The improvement in muscle power over the course of a month was dramatic. There was near-complete recovery of power of the left deltoid, biceps and triceps muscle with recovery of the left biceps reflex.

There are several outstanding observations in our case. As expected, the MRI findings display pathology of the C6 and C7 nerve roots. However, all three trunks of the brachial plexus display hyperintensities. The clinical examination and EMG findings reflect involvement of the upper trunk and posterior cord of the brachial plexus. We explain this discrepancy by the high sensitivity of STIR-MRI for endoneurial edema within the nerve fascicle, as we explain in the Discussion section. It may be that immune-mediated brachial plexopathy is a far more diffuse process and hence the term radiculo-plexopathy may be more appropriate, suggesting some of the neural inflammation may be subclinical. Another surprising finding is the fact that inflammatory changes on MRI of the brachial plexus persisted for two months after the onset of the disease. We will explain the evolution of pathology and MRI changes in the Discussion section. The last unusual observation is the rapid improvement to near-recovery two months after disease onset. We believe that this recovery is not part of the natural history of the disease as the patient's condition was static for at least two months prior to treatment, there was evidence of edema of nerves with MRI imaging and the recovery was too quick and can only be explained by the rapid resolution of inflammation due to intravenous pulse steroid therapy.

## Discussion

In order to understand MRN, one needs to understand the concept of a "magic angle". This is a specific angle of 54.74 degrees, a root of the Legendre polynomial in the spherical harmonics of magnetic resonance imaging (MRI) acquisition. This angle arises as an artefact with short echo T1 times which highlights well-ordered unidirectional collagen fibers giving the false impression of suprascapular tendinitis. The magic angle is avoided by prolonging the T1 echo times to 70-100 milliseconds. With respect to T2 weight images, the dipole-dipole interaction is proportional to

$$3cos^{2}\theta -1$$

where $\theta$ is the angle between the dipole vector and the magnetic field. This dipole moment is minimized when

$$3cos^{2}\theta -1=0$$

The solution to this equation is $\theta =54.74^{^{\circ}}$. The tissue of interest is rotated by $54.74^{^{\circ}}$ to the magnetic field, T2 decay is prolonged, thereby increasing signal intensity. This way the magic angle is enhanced thereby highlighting connective tissue (collagen) [12].

Meanwhile, a nerve fascicle is a collection of axons bounded by the perineurium. The endoneurium, a low-protein fluid with long T2 property, is the fluid that surrounds the axons within the fascicle. Disease of the nerve increases endoneurial fluid volume and hence T2 signal intensity. With chronic fibrotic changes of the nerve, the endoneurial fluid is lost and the prolonged T2 property is lost [13].

PTS is a clinical diagnosis, with no definite surrogate marker, and requiring proficient knowledge in brachial plexus anatomy and expertise in electromyographic studies. The localization of lesions of the brachial plexus can be technically challenging and the differential diagnosis can be intricate especially in atypical cases, where other diagnoses such as vasculitis or atypical chronic inflammatory polyradiculopathy (CIDP) need to be entertained [14]. The recent advances in MRI technology have been instrumental in helping the physician disentangle the complexities of the diagnostic process. MRN refers to a combination of imaging sequences including T1-weighted, T2-weighted fat-saturated sequences and STIR images that can outline the nerves from the spinal roots along the trunks and cords of the brachial plexus to the individual peripheral nerves (Table 2).

**Table 2:** Correlation of MRI findings and pathology. STIR: Short tau inversion recovery, MRI: Magnetic resonance imaging.

	NORMAL NERVE	INFLAMED NERVE
T1-WEIGHTED IMAGES	isointense to muscle	isointense to muscle
T2-WEIGHTED IMAGES	isointense to muscle	thickened; hyperintense compared to muscle
STIR IMAGES	slightly more hyperintense than muscle	thickened; hyperintense compared to muscle

In a study of 15 patients with the PTS, the suprascapular nerve, upper trunk of the brachial plexus, axillary nerve and long thoracic nerves were the most commonly affected nerves. The spinal nerve roots, trunks and cord of the brachial plexus and peripheral nerves were affected in descending order of frequency, the range being approximately 13-53%. The cervical C5 nerve root was the most frequently affected spinal nerve root [11]. In a review of 21 patients with PTS studied with T2-weighted MRI for high intensity signals, the suprascapular nerve was involved in 97% of cases and the axillary nerve in 50% of cases. Atrophy was noted in 30% of shoulders [15].

Muscle edema develops within 24-48 hours after denervation while electromyographic findings may take up to 7-14 days. Chronic denervation with atrophy and fatty replacement takes several months. Therefore radiological imaging can provide time-sensitive information [16]. In a retrospective survey of 26 patients with PTS, the most frequently involved and studied muscles around the shoulder girdle included the supraspinatus, infraspinatus and deltoids. Acute and chronic changes including intramuscular edema, atrophy and fatty infiltration and their evolution are listed below (Table 3) [10].

**Table 3:** MRI evolution of pathology of muscular denervation. MRI: Magnetic resonance imaging.

	T1-WEIGHTED IMAGES WITH FAST SPIN-ECHO	T2-WEIGHTED IMAGES WITH FAST SPIN-ECHO AND FAT SATURATION	PROTON DENSITY IMAGES	FAT-SATURATED OR T2 INVERSION RECOVERY IMAGES
INTRAMUSCULAR EDEMA		Acute: diffuse increase		
MUSCULAR ATROPHY	Chronic: shrinkage	Chronic: shrinkage		
FATTY INFILTRATION	Chronic: increased linear signals		Chronic: increased linear signals	Chronic: reduced signal

## Conclusions

Our case demonstrates the power of recent MRI technology in diagnosing and prognosticating disease of the brachial plexus. MRI-STIR positivity of nerves implies increased endoneurial fluid and inflammation and the potential for immunotherapy to be effective. Chronic changes such as fatty replacement may portend a worse prognosis; however, the presence of hourglass constrictions may offer the opportunity for surgical intervention. The blending together of the history, clinical examination, electrophysiological studies with the recent spectacular enhanced imaging technology opens up the opportunity for far better patient management, as we demonstrated serendipitously in our case. Further studies applying these new technologies are definitely warranted.
